# The Duration-Adjusted Reliable Change Index: Defining Clinically Relevant Symptom Changes of Varying Durations

**DOI:** 10.1177/10731911231221808

**Published:** 2024-01-27

**Authors:** Marieke A. Helmich

**Affiliations:** 1 Department of Psychology, University of Oslo, Oslo, Norway; 2 University of Groningen, University Medical Center Groningen, Interdisciplinary Center Psychopathology and Emotion regulation, Groningen, The Netherlands

**Keywords:** reliable symptom change, repeated measurement, within-person, clinically meaningful change, routine outcome monitoring, measurement-based care, transition duration

## Abstract

The time period over which relevant symptoms shifts unfold is not uniform across individuals. This article proposes an adaptation of the Reliable Change Index (RCI) to detect symptom changes of varying durations in individual patients’ time series: the Duration-Adjusted RCI (DARCI). The DARCI proportionally raises the RCI cut-off to account for its extension over additional time increments, resulting in different DARCI thresholds for different change durations. The method is illustrated with a simulation study of depressive symptom time series with varying degrees of discontinuity and overall mean change, and four empirical case examples from two clinical samples. The results suggest that the DARCI may be particularly useful for identifying symptom shifts that appear relatively abrupt, which can help indicate when a patient is showing significant improvement or deterioration. Its ease of use makes it suitable for application in clinical contexts and a promising method for exploring transitions in psychiatric populations.

## Background

Identifying whether and when a patient’s psychological symptoms have changed in a clinically relevant way is an integral part of many treatment settings, and studies of therapeutic interventions. Typically, methods to determine clinical change identify when a patient reaches a score over or under a certain threshold (e.g., within the range of a nonclinical population norm score; Jacobson et al., 1999) or shows a change in scores that meets a cut-off (e.g., 50% reduction; Ilardi & Craighead, 1994) or a combination of criteria (e.g., minimal score reduction and statistical significance; Jabrayilov et al., 2016). The Reliable Change Index (RCI) is a widespread method that determines whether the variability in a person’s measurements on a symptom questionnaire is more likely to be due to the instrument’s precision (measurement error) or due to an actual clinical change (Jacobson et al., 1999; Jacobson & Truax, 1991; Maassen, 2000; Maassen et al., 2009). Determining whether a symptom change indicated by a questionnaire is reliable is important to establish whether a drop or increase in scores is not merely due to chance and can aid decisions on whether to start, continue, end, or alter the intensity of treatment (Bauer et al., 2004).

As routinely measuring (former) patients’ psychological complaints and collecting time series of repeated assessments within individuals have become common practice in the context of therapy and relapse prevention (Boswell et al., 2015; Fortney et al., 2017; Lambert et al., 2018; Lewis et al., 2019; Schiepek et al., 2016), studies mapping repeated symptom assessments have shown that psychopathological change is often characterized by nonlinearity and abrupt changes (Gelo & Salvatore, 2016; A. M. Hayes et al., 2007; Helmich, Wichers, et al., 2020; Schiepek, 2009). Clinically, this is relevant as particularly sudden shifts may indicate that the patient may have experienced a transition to a better or worsened state, which could be predictive of their treatment outcomes (Aderka et al., 2012; Aderka & Shalom, 2021; Helmich, Wichers, et al., 2020; Shalom & Aderka, 2020; Tang & DeRubeis, 1999; Tang et al., 2002). Sudden gains (symptom improvements), for instance, may occur when a therapy session or intervention has been especially effective (Abel et al., 2016; Lutz et al., 2009; Shalom & Aderka, 2020; Tadić et al., 2010), while sudden losses (deteriorations) indicate when a patient is less likely to benefit from treatment, and should be identified as soon as possible to prevent the treatment from failing (Lutz et al., 2013; Thompson et al., 1995). A pattern of steady early improvement over the first few treatment sessions has also been linked to better treatment outcomes (Finch et al., 2001; Haas et al., 2002; Lambert, 2005; Lutz et al., 2009; Tadić et al., 2010), and conversely, early changes that did not conform to the expected response patterns have been linked to poorer outcomes (Lambert et al., 2002). Thus, identifying discontinuous symptom changes as they unfold is relevant given their association to treatment outcome.

Within-person change patterns of varying duration and magnitude have been described in the psychotherapy context (Rubel et al., 2015; Schiepek et al., 2017; Vittengl et al., 2016), but exploring a range of relevant time periods with existing methods is challenging. Most reliable change methods do not incorporate the time frame over which a change occurred, nor are they optimized for application to repeated symptom assessments. Many approaches are primarily used to test pre- to post-treatment change, which can take weeks or months, whereas others focus specifically on symptom shifts as they occur between or even within therapy sessions (Keller, 2003; Lutz et al., 2013; Tang & DeRubeis, 1999). For instance, the aforementioned sudden gains and sudden losses in symptoms are identified as changes between therapy sessions that combine a predefined minimum magnitude of change with the requirement that it takes place within a short period of time, most often a week (Aderka et al., 2012; Shalom & Aderka, 2020; Tang & DeRubeis, 1999; Tang et al., 2002). Yet, symptom reductions of 50% or more over 3 to 4 weeks of treatment have also been described as “rapid” in studies of early response and are certainly considered clinically relevant (Haas et al., 2002; Ilardi & Craighead, 1994). Symptom shifts may thus take different amounts of time and still be considered relatively abrupt, and this variability should be taken into account when studying periods of notable improvement, as the time it takes to change can be clinically meaningful in itself (Paul et al., 2019; Stulz et al., 2007).

Examining changes with a variable time frame and a standard single cut-off is not straightforward. Imagine two individuals who show the same reliable reduction in scores, for example, –15 points. For Person A, the cut-off is met relatively suddenly, from 1 week to the next, while Person B shows slower, gradual improvement and meets the same reduction only after 3 weeks. These individuals both show a reliable improvement according to this criterion, but due to the different timings, the process appears as a sudden change for one (Person A) and a much slower gradual improvement for the other (Person B). Here, using a single criterion is effective at detecting a minimally relevant reduction for both people, but it disregards the qualitative difference in “velocity” of the changes. Alternatively, one could set the threshold to apply to a fixed time frame such as 1 week and examine change over longer periods by requiring that the cut-off is met again each additional increment—in this case, change over 2 weeks would require a 30-point decrease. However, this approach lacks sensitivity to the fact that consecutive observations are being considered. To illustrate this point further, imagine the stepwise changes shown by a third person, Person C, who shows a pronounced decline in scores over multiple weeks (e.g., steps of −12, –9, –11 points). The changes between two adjacent points (over 1 week) never meet the minimal change criterion of −15 points, yet the overall change shown by person C is substantial: –32 over the 3 weeks, more than double the 15-point threshold. Intuitively, one could also consider this pattern a clinically relevant and rapid improvement (Haas et al., 2002; Ilardi & Craighead, 1994) and one worth detecting. However, as illustrated, using a single cut-off that is unadjusted for the duration of a symptom change allows one to determine reliable change over a set interval (Person A) but will miss smaller within-week changes that culminate into a relevant change over a longer period (Person C). Ideally, a change criterion would be able to account for clinically meaningful changes of different durations by requiring that changes over longer periods must also be larger as a whole, although not necessarily so large as a basic multiplication of the cut-off. Then, Person C’s consistent improvement could be identified as a reliable change that is comparable in its clinical relevance to the rapid 1-week shift shown by Person A. In short, standard available single cut-offs cannot optimally identify clinically relevant changes that occur over consecutive time points.

To summarize, various methods exist to determine whether a relevant change in symptoms has occurred at the within-person level, but these typically make no particular assumption about the time it took for the symptoms to change. Furthermore, even those that examine sudden gains and losses in repeated assessment data do not provide solutions to identify abrupt changes that extend over multiple time points or therapy sessions. More data-intensive methods may focus on testing the significance of an overall symptom change with a regression model (Ferrer & Pardo, 2014; Maassen et al., 2009; Maric et al., 2015; Slofstra et al., 2018) or try to identify abrupt shifts with a change-point model (Albers & Bringmann, 2020; Cabrieto et al., 2017), but these methods may be difficult to implement in real time in the course of clinical practice, as they require more data than may be available in early stages of treatment, and also a fair level of statistical knowledge to be conducted (de Vries & Morey, 2013). Thus, adjusting a simple method like the RCI may help to identify both reliable symptom changes that are very abrupt and symptom transitions that accumulate into considerable change over a slightly longer time frame.

In this article, I propose a method, based on the well-established RCI (Jacobson & Truax, 1991), which allows researchers and clinicians to explore the presence of symptom changes of varying durations in individual patients’ time series: the Duration-Adjusted Reliable Change Index (DARCI). A simulation study is conducted to test the DARCI’s ability to pick up periods of relevant change, and discontinuous change in particular, in the context of a larger overall symptom time series. Empirical case examples from two clinical data sets, with visualizations of the DARCI’s detection of transitions at different confidence levels (CLs), are also presented.

## Method

### Materials

This study uses the Symptom Checklist–90 (SCL-90) depression subscale and Dutch norm scores (Arrindell & Ettema, 2003; Derogatis, 1977) as a basis for illustration of the DARCI in the simulation study and in the first set of empirical case examples. This questionnaire consists of 16 items that ask to what extent one was bothered in the past week by particular depressive symptoms (e.g., “feeling blue”) on a 5-point scale ranging from *not at all* to *very much*. The second set of empirical case examples uses the Beck Anxiety Inventory (BAI; Beck et al., 1988), which is a 21-item self-report instrument to assess the severity of anxiety symptoms (e.g., “fear of the worst happening”) in the past week on a 4-point scale ranging from *not at all* to *severely*.

### Reliable Change Index

The RCI was developed as a method to ensure that any identified pre- to posttreatment change was a reliable change that could be distinguished from measurement error (Jacobson et al., 1999; Jacobson & Truax, 1991). The RCI can be used to calculate a threshold at which the difference between a pre- and postmeasurement for one person is, with a 95% two-tailed CL, “unlikely to occur without actual change” (Jacobson & Truax, 1991, p. 14). It uses the standard error of measurement ( ${SE}_{m}$ ) from a population norm of a given instrument to calculate the minimum score necessary to exceed a change that could be due to measurement inaccuracy ( ${SE}_{\mathrm{diff}}$ , standard error of difference). Note that the RCI uses between-persons information for the standard error of measurement^1^ regarding the spread of the distribution of test–retest reliability given no change and uses this to test whether within-person changes are reliable. It is defined as follows:

(A.5) $$RCI={SE}_{\mathrm{diff}}\times Z,\;\mathrm{and}\;{SE}_{\mathrm{diff}}=\sqrt{2{\left({SE}_{m}\right)}^{2}}.$$

For this study, ${SE}_{m}=4.37$ , taken from the Dutch SCL-90 depression subscale, based on the psychiatric outpatient norm group of 5,621 patients (cf. SCL-90 manual; Arrindell & Ettema, 2003). Thus, ${SE}_{\mathrm{diff}}=\sqrt{2{\left(4.37\right)}^{2}}=6.18$ , and $\mathrm{RC}I_{95}=6.18\times 1.96=12.11$ . Where 1.96 is the *Z*-score for a 95% CL, and a score of ~13 is a reliable change between two observations on the SCL-90 depression subscale. The score is rounded up to ensure the CL is maintained, as the (difference) scores are always integers, thus obtaining a score of 12.11 is not possible.

### Duration-Adjusted RCI (DARCI)

The DARCI is proposed as an adaptation of the RCI, with the aim to capture symptom changes of varying durations, particularly changes that appear as sudden or large in overall scope. The DARCI requires setting a fixed time period between two points (e.g., 1 week) as a basis for extension when more points are added (i.e., when testing change over longer durations) and allows one to calculate thresholds for each additional increment of time (e.g., each added week). Like the regular RCI, the DARCI tests the difference score between two points, but it accounts for instances where the two compared points are farther apart by proportionally increasing the change threshold.

To detect reliable change from a given starting point (*t*_start_) to the last observation in a chosen period (*t_n_*) while considering the additional time between these observations, the original RCI threshold (based on *n* = 2 observations) is divided by 2 and multiplied by the number of observations in the range of interest. To calculate the DARCI critical change threshold for a particular *Z*-value (confidence level) and number of observations ( n ),

(A.5) $$DARCI=\left(\frac{{SE}_{\mathrm{diff}}}{2}\right)\times Z\times n.$$

Essentially, the RCI threshold is reduced to a range of uncertainty around a single point, and then proportionally extended for the number of observations (i.e., period of time) at hand while maintaining the chosen CL (e.g., 95%). In doing so, the DARCI provides a way to detect symptom changes over various durations with the same degree of reliability, even if some shifts take longer. Applied to our illustrative sample and instrument, with the ${SE}_{\mathrm{diff}}$ for the SCL-90 depression subscale incorporated: DARCI=(6.18/2)×Z×n. For 95% confidence, this can be rewritten as ${DARCI}_{95}=3.09\times 1.96\times n=6.06\times n.$ , where 6.06 is the aforementioned range of uncertainty around one point.

Alternatively, we can calculate the *Z*-score for a particular change over time (i.e., the difference between two assessments Δy ), divided by the number of observations ( n ) in the range of interest. This can be useful to compare the relative magnitude of multiple identified changes. In formula form,

(A.5) $${DARCI}_{Z}=\frac{\left(2\frac{\Delta y}{n}\right)}{{SE}_{\mathrm{diff}}}.$$

The DARCI thresholds for different increments are presented in Table 1. Using this method, higher and lower CLs may also be calculated and explored, and all DARCI critical threshold scores are rounded up to maintain the cut-off ≥*Z*-score requirement.

**Table 1.** The DARCI Over Different Durations and Confidence Levels for the SCL-90 Depression Subscale.

Thresholds	*n* = 2	*n* = 3	*n* = 4	*n* = 5
	*1 week* ^*a*^	*2 weeks* ^*a*^	*3 weeks* ^*a*^	*4 weeks* ^*a*^
*Z*≥1.65 (90% CL)	10.17 ≈ 11	15.25 ≈ 16	20.33 ≈ 21	25.42 ≈ 26
*Z*≥ 1.96 (95% CL)	12.11 ≈ 13	18.17 ≈ 19	24.23 ≈ 25	30.28 ≈ 31
*Z*≥ 2.58 (99% CL)	15.94 ≈ 16	23.92 ≈ 24	31.89 ≈ 32	39.86 ≈ 40

*Note*. To calculate the cut-off scores, the following formula was used: $DARCI=\left(\frac{6.18}{2}\right)\times Z\times n$ , where n is the number of observations over which the change occurs. The scores are rounded up to maintain the required minimum *Z*-score. Change between two observations is equal to the original RCI. DARCI = Duration-Adjusted Reliable Change Index; SCL-90 = Symptom Checklist–90; CL = confidence level; RCI = Reliable Change Index.

a The indicated time periods are illustrative, to show that the threshold is extended proportionally over time increments of equal size. This could also be (a number of) days or another set number of weeks.

The DARCI does not prescribe over which time period the change must take place. Instead, the time interval between two observations serves as a basis for the other increments over which it is extended. Researchers or clinicians must choose the duration of time over which detecting a change would be of interest (e.g., based on the clinical literature, pilot studies, or other conceptual grounding). For instance, if the change between *t*_start_ and *t*_2_ occurs over 1 week, then the DARCI will be proportionally extended for 2, 3, 4 weeks, and so on (as the limits of a given scale allow); if *t*_start_ to *t*_2_ occurs over 2 weeks, reliable change can be calculated for 4, 6, 8 weeks, and so on. It is thus an important theoretical and clinical consideration what durations of change are relevant to capture, although the DARCI lends itself precisely to exploring a number of different possibilities.

### Analysis

#### Simulation Study

To test the accuracy of the DARCI thresholds for change over two (Tn2), three (Tn3), and four (Tn4) time points, the frequency at which modeled shifts were correctly identified in simulated repeated symptom assessments was examined. A set of 10,000 time series with a length of 15 points was simulated, to reflect a typical duration of psychological treatment (Gloaguen et al., 1998; Hansen et al., 2002). Each time series was drawn from a randomized normal distribution with a mean of 0 and variance concurrent with the SCL-90 depression subscale ${SE}_{m}$ of 4.37. The time series were then fitted to different overall symptom reductions (L = −10, –20, –30, –40, which correspond to 16%, 31%, 47%, and 63% of the total scale, respectively) and different degrees of discontinuity. Specifically, five shapes of increasingly abrupt change around the midpoint of the time series were modeled: (a) gradual change as a linear function (no internal shift); (b) a smooth curve formed by a sigmoid function with a slope of 1 at the origin (k), and the midpoint set to point 7.5 (the steepest decline occurs between Observation 7 and 8); (c-e) three mean shifts where change takes place over an increasingly short time period: (c) a step-function with the decrease occurring over four points (*n* = 4); (d) a step-function with the decrease occurring over three points (*n* = 3); and (e) a step-function with the shift occurring from one point to the next (*n* = 2).

These change patterns were chosen to explore the interplay between the strength of the overall slope and different degrees of discontinuity. For instance, at an overall reduction of 10 points and a linear function, any identified change would be due to a random fluctuation, as the (DA)RCI thresholds start at 13 points for *n* = 2. At the other extreme, at −40 points of overall change, we would expect that, even if the discontinuities are more gradual (like in the sigmoid function, or the step-change over four points), the DARCI thresholds will often detect the modeled shifts around the correct time period. Furthermore, three of the five models are step-functions, which allow certainty about when a shift starts and ends, as this is explicitly modeled. This is in contrast to the sigmoid curve, which may represent a more naturalistic change pattern. Moreover, the durations of those shifts correspond to the different durations of the DARCI thresholds that were tested, over two, three, and four points, and should thus provide a good test of the thresholds’ sensitivity.

In short, DARCI thresholds at 95% CL were calculated for change over two (Tn2), three (Tn3) and four observations (Tn4) and applied to simulated symptom time series to identify periods over which the criteria for a reliable change were met. Particularly, we examined the sensitivity of the DARCI thresholds for finding the modeled discontinuities in the middle of the data set, and the extent to which they were specific to indicating the intended shift, rather than random fluctuations.

**Figure 1.**
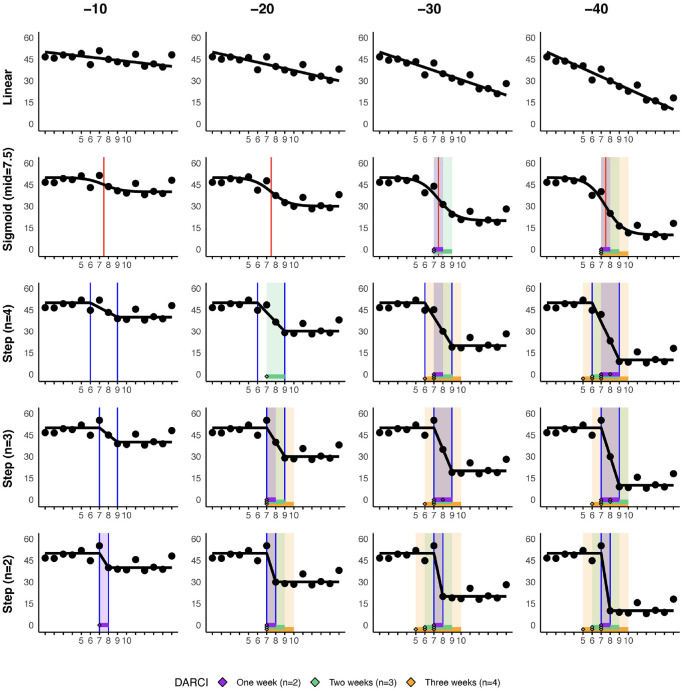
Case Demonstration of a Simulated Time Series, With Increasing Levels of Overall Decline, and Increasingly Abrupt Shifts Around the Middle of the Time Series. *Note.* Increasing levels of overall score reductions (–10 to −40) are shown on the general X-axis (see headers) and increasing discontinuity around the midpoint on the general Y-axis (from a linear model without a shift, to an abrupt step-change between two points). Periods where reliable changes were indicated by the DARCI (with durations between 1 and 3 weeks) are shown with the colored areas (i.e., purple, green, orange), with the point markers indicating the start of a reliable change. The red vertical line indicates the simulated midpoint of the sigmoid pattern, and the blue vertical lines indicate simulated start and end points for the step-function. DARCI = Duration-Adjusted Reliable Change Index.

**Figure 2.**
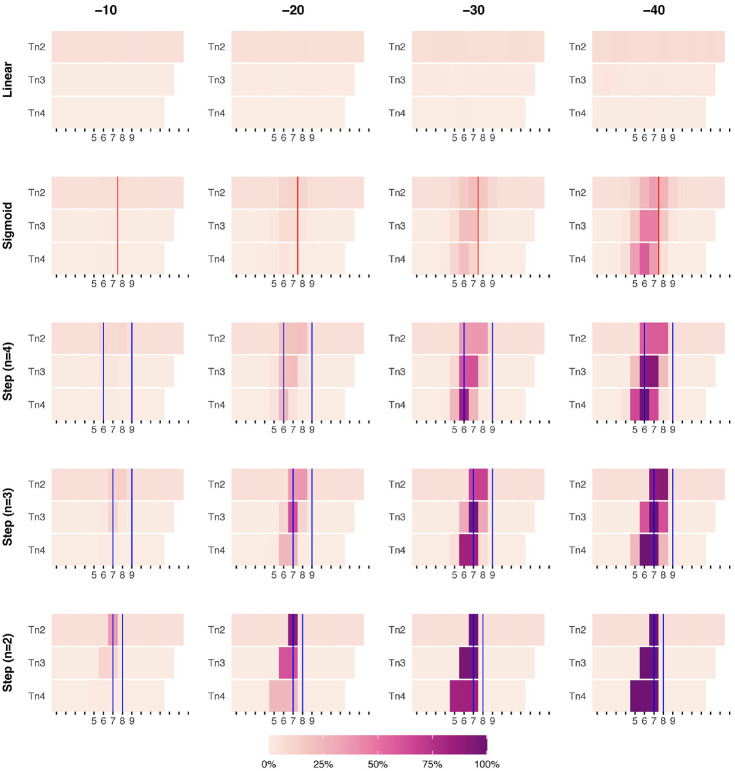
Heatmap of DARCI-Detected Starting Points (t_start_) of Reliable Changes in the Simulated Time Series. *Note.* Visualization of the frequency of indicated starting points of reliable change across the 1,000 simulations. Varying levels of overall change (−10 to −40) are shown on the general X-axis (see headers) and increasing discontinuity on the general Y-axis (from no shift within the time series, to an abrupt shift between two points). The red vertical lines indicate the midpoint of the sigmoid function, and the blue lines indicate the start and end of the step-functions. Tn2 = (DA)RCI calculated for change between two points; Tn3 = DARCI calculated for change over 3 points; Tn4 = DARCI calculated for change over 4 points. Note that the location of *t*_start_ contributes to the value count, and the accuracy of the DARCI must be inferred from whether the simulated change period falls within the range of the threshold’s duration (Tn2, Tn3, and Tn4). DARCI = Duration-Adjusted Reliable Change Index.

**Figure 3.**
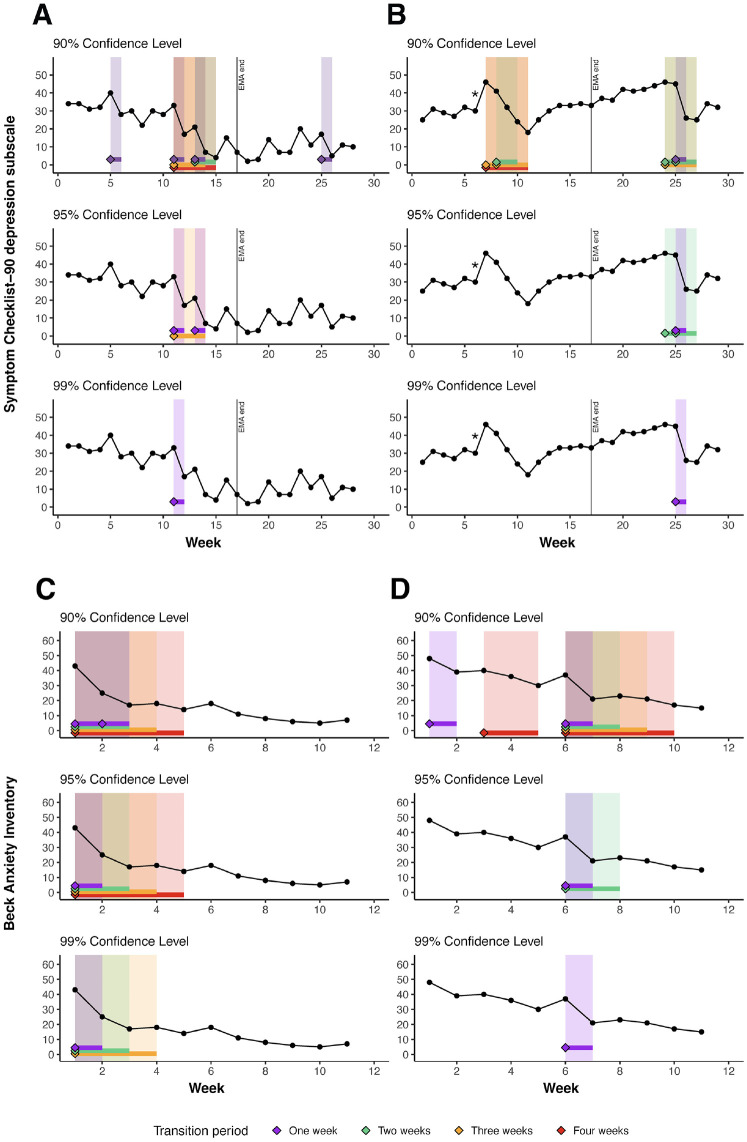
Application of the DARCI Across Confidence Levels (90%, 95%, 99%) in Four Empirical Case Examples. *Note.* Cases A and B are individuals from the TRANS-ID Recovery data set who completed 14 of the 16 items from the Symptom Checklist–90 depression subscale every weekend over the course of 6 months, during which they received psychological treatment for depression. The data collection also included 4 months of EMA, the end of which is indicated by a vertical line. Cases C and D are individuals who received treatment for anxiety at Modum Bad psychiatric hospital and completed the 21-item Beck Anxiety Inventory at the beginning of every week during the period they received treatment. DARCI = Duration-Adjusted Reliable Change Index; EMA = ecological momentary assessment.

All analyses were conducted in R (version 4.3.0), and code for the simulations and plotting of the symptom data (Figures 1–3) is available online at https://osf.io/24cfa/.

#### Empirical Case Examples

The application of the DARCI in clinical data is illustrated by visualizing the identified reliable *improvements* found in the symptom time series of two individuals from the following two data sets. To preserve the anonymity of the chosen cases, no details are shared about the persons’ age, gender, diagnosis, or treatment.

Two cases were drawn from the TRANS-ID Recovery data set (Helmich, Snippe, et al., 2020), an intensive longitudinal study of individuals receiving psychological treatment for depression, who completed an average of 23 weekly SCL-90 depression subscale (Arrindell & Ettema, 1981; Derogatis, 1977) measurements during the 6-month assessment period. The DARCI has previously been applied to these data in a study that focused on detecting changing dynamics in ecological momentary assessments prior to the identified symptom transitions (Helmich et al., 2023). Note that in the weekly measurements of this data set, two items on suicidal ideation were omitted from the 16-item scale, which means that the calculated DARCI thresholds are likely to yield more conservative results in this sample.

The second set of cases was drawn from anonymized patient data from the Modum Bad psychiatric hospital anxiety department (see also Johnson et al., 2017). Weekly BAI assessments are collected as a standard part of care during psychological (inpatient) treatment, about 10 assessments per person during therapy. The example cases were taken from a random subset of 50 cases that belong to a larger pool of data collected between 2016 and 2023. The RCI calculation for the BAI is based on the original research by Beck et al. (1988), as there are currently no representative psychiatric norm scores available for the Norwegian population (Lisøy & Martinsen, 2023) and the same RCI has been used in previous studies on these data (Johnson et al., 2017). Based on a reliability (Cronbach’s α) of .92, and a consequent^2^ ${SE}_{m}$ of ∼3.25, the ${SE}_{\mathrm{diff}}$ = 4.59, and the $DARCI=\left(\frac{4.59}{2}\right)\times Z\times n$ .

## Results

### Simulation Case Illustration

A visual illustration of the ability of the DARCI to identify the intended periods of relevant change is provided in Figure 1. The indicated changes for the simulated time series in Figure 1 demonstrate clearly how the DARCI was able to pick up on the periods of increased discontinuity, and thus, relevant change—especially when overall change was at least 30 points. At a lower overall reduction, of 20 points, only the more discontinuous changes were detected in this case example.

Another noteworthy point is that thresholds for reliable changes of different duration may be met simultaneously. This can be seen, for instance, in the bottom row of plots, Step (*n* = 2) in Figure 1, where change is modeled between two points, but it was large enough that the DARCI over three (*n* = 3) and four points (*n* = 4) also picked it up as reliable. The meeting of a threshold is not inherently precise: We see that with the Tn4 cut-off and duration, we are unable to pinpoint the location of the modeled narrow shift, and multiple starting points (x = 5, 6, 7) are indicated. This is in contrast to the DARCI over two points, which naturally identifies the shift at a single location (between Observation 7 and 8). The reverse also happens, where the DARCI over two points cannot precisely identify the location of a shift in the step-function over *n* = 3 and *n* = 4, and thus places multiple starting points (x = 6, 7, 8) in these longer change periods. Apart from visual inspection, the best fitting duration for any identified shift can be determined by comparing the *Z*-scores. The relative strength of a shift over a given duration can be calculated, with higher absolute values indicating which duration and time period best describe an identified transition. For instance, the aforementioned shift, Figure 1, –40, Step (*n* = 2) has a *Z*-score of −7.3 when the DARCI is calculated for the change over two points (Tn2), and *Z* = −5.0 at Tn3, and *Z* = −3.7 for the Tn4 change, and is thus clearly best described by the 1-week (Tn2) transition period.

### Results of the Simulation

In Figure 2, the results of the 10,000 simulations are visually represented as a heatmap, with higher values (darker purple) representing a higher frequency of DARCI-indicated change start points for different durations (Tn2, Tn3, and Tn4).

#### Linear

Looking at the first row of subplots, for Linear change, we see a low degree of false positives across the different thresholds. There is a slightly higher degree of false positives for the DARCI threshold for changes between two points (Tn2), indicating that the criterion picked up approximately 5% to 7% random fluctuations rather than true changes—this also applied to the DARCI for change over two points in the other overall change models. The DARCI criteria for changes over three or four points showed negligible rates of false positives across the overall change levels, 0.5% to 2% at Tn3% and 0% to 0.6% at Tn4.

#### Sigmoid

Looking at the Sigmoid change curves, where the exact starting point of change is less apparent, the DARCI method started to noticeably pick up changes from an overall change of −30: about 21% for all three different duration thresholds. When the overall amount of change was −40, the DARCI thresholds identified the correct period of increased discontinuity in about 35% to 58% of the simulations.

#### Step (n = 4)

For the third row of subplots in Figure 2, changes started to be noticeably detected in the −20 overall change model. Given that the change was modeled to take place over four time points, the Tn4 threshold yields the highest specificity: 24% of changes were determined to start at Time Point 6, as modeled. At higher levels of overall change, this increased to 82% and 100%. This while in the −20 overall change models, changes over two (Tn2) and three points (Tn3) were still similar in accuracy: About 19% and 21% of simulations identified a change in the correct period, respectively. For Tn2, this rose to 37% at −30, and 58% at −40 overall change, whereas Tn3 accurately captured 62%, and 91% at those levels of overall change.

#### Step (n = 3)

When change was modeled as a step-function over three time points, the DARCI over three change points picked this up accurately at 62% at −20 points overall change, and 97% and 100% at larger changes (−30, −40). To compare, the DARCI at Tn2 showed about 37% accuracy at identifying the location of the mean shift at −20, but the identified starting points were placed at Time Points 7 and 8, as the 1-week duration could not capture the entire change period that was modeled. Conversely, for the longer duration of Tn4, the changes were harder to identify because often the overall required change (Tn4 = 25) to meet that threshold was not met (only in about 24% of cases at −20 decline). The detection rates for these durations improved when the overall change increased to −30: with about 68% of simulations indicating changes over two time points, and 82% finding shifts over four points as well. At −40, the Tn2 threshold picked up the modeled shift period 90% of the time, and 99% with the Tn4 criterion. Worth noting is that the transition thresholds of Tn3 and Tn4 are occasionally also met for x-values that lie before or after the actual modeled shift. For instance, Tn3 is met 61% of the time at both x = 6, and x = 8 when the overall change is −40, because part of the modeled shift was already sufficiently large to meet the threshold. This “blurring” effect is visible in all the modeled shifts that occur over more than two time points (from Sigmoid to Step *n* = 3).

#### Step (n = 2)

In the bottom row of subplots, where a mean shift was modeled as occurring between two time points, the RCI was still able to pick up the correct location of the shift for about 36% of cases at Tn2 and for about 9% at Tn3 at the lowest level of overall score reduction (−10). For the −20 change model, the DARCI over two points picked up the change point accurately in 90% of simulations (compared with 62% at Tn3, and 24% at Tn4). At −30 overall change, only the Tn4 model did not always pick up the modeled shift (the other thresholds picked up ∼100%), yet in 82% of simulations it indicated a shift started somewhere in the range of time point 5 to 7. Finally, at the highest level of overall change (−40), the change was correctly identified in 100% of cases for all DARCI thresholds.

### Empirical Case Examples

Figure 3 shows the results of the four empirical case examples from the depression (A and B; TRANS-ID Recovery) and anxiety samples (C and D; Modum Bad). Per case, three subplots are presented that demonstrate the DARCI-identified *improvements* from 90% to 99% CL in their symptom time series. The information in those subplots naturally overlaps, and with the increasingly strict CL fewer shifts will be marked as meeting the cut-offs. By looking across all cases, we may get an impression of the presence of relevant shifts of varying durations in real clinical data, and of the comparative sensitivity of the DARCI thresholds and CLs at picking up these changes. These empirical cases are selected for their suitability to illustrate the method, with other patients in the data sets showing similar or more complex patterns of shifts, but some also lacking any detected changes.

#### Case A

In the first empirical case example, we see a depression symptom time series with an overall improvement (from moderately high to mild symptoms) and relatively many fluctuations from week to week. We can see that the 90% threshold picks up seven shifts in total, where smaller fluctuations also meet the cut-off. Note that some of the weekly fluctuations toward higher symptom levels likely also meet these criteria. The smaller changes are no longer picked up in the second subplot, as we see the first and last 1-week (purple) transitions disappear once the confidence level is raised to 95%, and the 2-week (green) and 4-week (red) period, which both include a very minor score change from Week 14 to 15, no longer meet their respective DARCI thresholds. The cascade of two large decreases from Week 11 through 14 is captured well at 95% CL, with two 1-week and a 3-week (orange) shift. However, at the strictest CL of 99%, only the largest 1-week shift remains reliable.

#### Case B

The depression time series of the second case example generally shows smaller incremental steps from each week to the next, no persistent change from the moderately severe symptom levels, although it is notably marked by two phases of discontinuity in the trajectory. There is an interesting period of continued gradual improvement from Week 7 through 11, which is preceded by a deterioration (indicated by the *asterisk) which is reliable at the 99% CL. The improvement is marked by the DARCI thresholds at 90% CL: in its entirety by a 4-week transition, as well as two 3-week and one 2-week shift. This period is no longer identified as reliable at higher CLs, and the fact that it is preceded by such a strong deterioration may also alter the clinical evaluation of the improvement. Later in this time series, we see a clear demonstration of overlapping transitions being separable by *Z*-score. The 1-week transition at Week 25 is the only remaining reliable shift at 99% CL, which is indicative of its relatively larger *Z*-score. At the lower CLs, the overlapping transitions of longer duration show a “blurring” around this change, as the 2-week changes place a starting point at both Week 24 and 25, although the scores from 24 to 25 and 26 to 27 that they include in that period have negligible decreases.

#### Case C

Moving to the anxiety symptom time series collected during treatment, Case C represents a patient who improves over the course of 11 weeks from severe to mild symptom levels, with the most marked improvement occurring in the first 4 to 5 weeks. The change across those first weeks, as maintained at 99% CL for 1-, 2-, and 3-week durations, appears to be driven by the strong symptom drop from Week 1 to 2, particularly considering that the overall change remains reliable despite the slight increase in scores from point 3 to 4. Examining the *Z*-scores for those transitions reveals some of the weighting effect of the DARCI: Tn2 = −3.92, Tn3 = −3.77, Tn4 = −2.72, and Tn5 just misses the 99% reliability cut-off with −2.53. Although the 2-week period is almost as strong as the 1-week change, note that without adjustment for the number of time points, the change from point 1 to 3 would have had a *Z*-score of −5.66. By considering the fact that most of the change happened over 1 week, we can evaluate cumulative, built-up changes, as illustrated here.

#### Case D

This final empirical case shows an overall trajectory from severe to more moderate symptom levels during treatment. Again, we see that the most profound change is a 1-week shift, here located between Weeks 6 and 7. The time series shows various periods of improvement identified by the DARCI thresholds at 90%, but these also illustrate the importance of exploring the appropriate level of sensitivity, as most of these changes are not maintained at higher CLs, and yet many other patients never meet any of the thresholds even at the 90% CL.

## Discussion

This article provided a first illustration of a newly proposed method to identify reliable changes in symptoms over multiple increments of time. Based on the simulation study and empirical case examples, it appeared that the DARCI was well-suited at picking up relevant (discontinuous) changes of varying length in the overall course of the symptom time series. Where transitions overlapped, the period that showed relatively (to the time it took) the largest change could be identified by looking at which of the overlapping transitions had the highest *Z*-score. All in all, the DARCI was demonstrated to be a useful extension of the standard RCI for detecting and exploring reliable shifts over multiple time points in the course of treatment or other repeated assessment applications.

The simulations showed that the DARCI thresholds generally were accurate and sensitive to changes, especially when the overall change was large (≥30 points, or about half the total scale). The DARCI thresholds over more than two observations (Tn3 and Tn4) were able to pick up the modeled points and periods of discontinuity with high accuracy, and the longer durations also showed fewer false-positive values than the DARCI for change over two points (Tn2, which is equivalent to the standard RCI, but set to a chosen time interval). The DARCI over three time points (Tn3) seemed to be particularly well-suited to identifying both the relatively abrupt changes modeled with the step-function over two and three observations, and the more gradual changes in the sigmoid function and the slowest step-function over four points. The DARCI over four observations (Tn4) also performed well, even though it showed slightly more dispersion of the identified start points. This “blurring” effect was not necessarily a sign of poor performance, as the modeled transitions did meet the Tn4 criteria, but the starting point of quicker changes was less precisely estimated due to the larger range of the DARCI at that duration. Similarly, the threshold for change over two points sometimes picked up reliable changes within the context of a larger overall shift, thus identifying a partial transition. Taken together, the simulations showed that the DARCI thresholds could accurately pick up known shifts and detect instances when a shift was less well-defined. Extrapolating these results to an applied context, we may be confident that the DARCI picks up larger changes very well, and that smaller changes could also be explored by lowering the CL.

Apart from the accuracy of the DARCI thresholds, the simulations also revealed that investigating reliable changes over different time periods with a purposely adapted index has the potential to uncover clinically relevant symptom changes that may be modest, step-by-step, but large overall. Employing only the standard RCI cut-off and testing difference scores without regard for time (any change that meets the criterion counts), or with a mere repetition of the same criterion for each increment (test the differences for point 1–2, point 2–3, etc., change is found only when those adjacent points meet the cut-off), would overlook these kinds of continued changes. This is further supported by the empirical case examples, which showed that the detected periods of reliable change often spanned multiple weeks, even if the largest change did tend to be a shift over a single week. A more extensive exploration of the prevalence of the various change durations is needed to understand the interrelation of these faster and slower transition processes more fully. Clinically, it is interesting to learn more about when and how multiple change thresholds are met by one individual, and broadening that, to explore which kinds of changes tend to occur within a given study population (e.g., response patterns in depression, Korf, 2014; Rubel et al., 2015; Vittengl et al., 2013, or mood shifts in bipolar disorder, Bos et al., 2022; Kramlinger & Post, 1996). Moreover, the DARCI method may provide a novel way to describe and explore within-person change patterns, which have shown to be of importance for the outcome of treatment (e.g., A. M. Hayes et al., 2007; Lutz et al., 2013; Schiepek et al., 2017; Stulz et al., 2007; Thompson et al., 1995; Vittengl et al., 2016).

The ability of the DARCI method to detect periods of relevant change relies on the chosen time period between observations, and setting an appropriate duration from which to extend the DARCI thresholds is quite the conceptual challenge. The time scale of clinical change processes is an ongoing and highly important topic of study in and of itself (S. C. Hayes et al., 2019; Kazdin, 2001; Mahoney, 2004; Strunk & Lichtwarck-Aschoff, 2019), so there is no readily available gold standard. Researchers must decide for their study what the relevant periods of change may be, which may require pilot studies and in-depth clinical knowledge of the population and change process under study to come to an educated best guess. In a clinical context, priority may be given to what is available, for example, session-to-session assessments. Yet, this uncertainty around the most appropriate time scale for the DARCI thresholds also offers grounds for exploratory studies. While more frequent measurements would likely reveal more nonlinearity over time (Helmich, Wichers, et al., 2020; Schiepek, 2009), there may nonetheless be practical limitations (e.g., patient burden) or theoretical considerations (e.g., conceptualization of symptoms; Bringmann et al., 2022) that lead one to prefer weekly intervals or simply observations between therapy sessions. The application of the DARCI to time periods ranging from days to months could be investigated to learn more about the optimal time scale to describe the process of symptom change that would be captured with this method.

Commonly, when more data are available, the additional power allows smaller changes to be detected (e.g., in a linear regression), so the fact that the change thresholds increase for longer periods may be unexpected from a purely statistical standpoint. While this is a fair observation, the aim is not to determine a statistically significant change (Bauer et al., 2004; Jacobson et al., 1999; Jacobson & Truax, 1991). Instead, the DARCI may provide a simple method to add to a clinician’s toolbox, which allows periods of reliable change to be uncovered within the course of a longer time series, even when little data are available. The DARCI uses between-persons information (the ${SE}_{m}$ from the instrument’s norm group) to allow within-individual changes to be differentiated from measurement error. Certainly, some individuals may still show variations far beyond the expected range, seemingly showing reliable changes almost every other step; just like some individuals are more likely to vary very little over time, and never show changes that meet the threshold, even if they experience them as meaningful. Once more data are available, more refined methods may be useful in determining significant individual change points or overall change (Albers & Bringmann, 2020; de Vries et al., 2014; de Vries & Morey, 2013), and future research may investigate the use of multilevel estimation for obtaining individualized DARCI thresholds.

This method is not without some limitations. First, the proposed method for extending the RCI is rather simplistic, which may mean that the thresholds for longer changes is not optimally tuned (or too strongly penalized) compared with change over two points. This might explain the predominance of highly reliable 1-week changes in the empirical case examples. However, given the alternative of using a single cut-off without any concession to time, this method still offers an improvement and a first step toward the in-depth exploration of within-person symptom changes. Second, although the DARCI is very flexible and can be adapted to any chosen symptom measurement instrument, the RCI relies on clinical norm scores for optimal performance (Jacobson & Truax, 1991). In our simulations and first set of empirical case examples, we used the SCL-90 norms as a reference as they are based on a large sample (*N* = 5,621). Other instruments may not have such norm scores available, as seen in the BAI of our second set of empirical case examples, which makes calculations of the standard error of measurement and consequently the (DA)RCI potentially less accurate (Bauer et al., 2004; Jacobson et al., 1999). It is also worth remarking upon the time period underpinning the ${SE}_{m}$ : the reliability coefficient on which the ${SE}_{m}$ is based is drawn from test–retest reliability (although internal consistency can be used as well), which would likely be based on a repeated assessment after several months (Bauer et al., 2004). This is quite different from the time periods (weeks) discussed in this article for the DARCI. However, this problem may be minor, as the chosen ${SE}_{m}$ is likely to have resulted in more conservative thresholds, as test–retest reliability would be higher if measurements occurred close together in time (and the ${SE}_{m}$ smaller). Finally, it is worth noting that DARCI thresholds may be met, even if there are moments of significant change in the opposite direction just before (Figure 3B) or within the period (e.g., slower transitions like Tn4) under consideration. For instance, a brief depression spike in an overall course of improvement. To account for such strong fluctuations and nonlinearities, it may be worth applying the DARCI to detect both improvements and deteriorations within the same time series, so that reliable shifts in either direction are picked up and their impact can be compared. In addition, the DARCI is a tool that can augment but not replace clinical judgment, which remains vital for interpreting and contextualizing the presence of meaningful symptom change.

A strength of this method is that it has the potential for easy application in the clinical context. Once the thresholds have been calculated, clinicians can check whether incoming new symptom assessments (e.g., as gathered with routine outcome monitoring) meet the shorter or longer duration thresholds of the DARCI. This has applications during treatment, to monitor the psychotherapy process, as well as afterward, in relapse prevention monitoring. Moreover, the nature of transitions in mental health is a topic that warrants further study and using this method to explore the presence of sudden-gain-type shifts as well as slower accumulated reliable change could yield novel insights into within-person psychological change processes.

Future research should further validate this method with clinical data from various patient populations and instruments, to provide insight into the kind of symptom changes that can be detected and expected in real-world symptom assessments. In addition, comparing the DARCI with existing models of change within the course of treatment, such as sudden gains (Tang & DeRubeis, 1999) would be worthwhile, although the two methods may have slightly different objectives (i.e., identifying changes of various durations vs. change between therapy sessions).

To conclude, the DARCI provides a simple adaptation of a well-established method for identifying reliable change (Bauer et al., 2004; Ferrer & Pardo, 2014; Jabrayilov et al., 2016) and may encourage researchers to consider exploring (discontinuous) symptom shifts of varying durations in the context of psychological treatment.

## Notes

1. Ideally, the same time frame is used for the calculation of the standard error of measurement (based on the test–retest reliability; Ferrer & Pardo, 2014; Maassen et al., 2009) as for the investigated difference score. However, in practice, this time frame is usually not considered and the RCI is calculated with the available information.

2. According to the formula $\mathrm{SD}\sqrt{1-r}$ .
